# Pyogenic Liver Abscess Growing Streptococcus constellatus in an Elderly Female With Recent Diverticulitis: A Case Report

**DOI:** 10.7759/cureus.37004

**Published:** 2023-04-01

**Authors:** Seyed Mohammad Nahidi, Murtaza Syed Hussaini, Devi Mahadeo, Zeyar Thet

**Affiliations:** 1 Internal Medicine, Wyckoff Heights Medical Center, Brooklyn, USA; 2 Medicine, St. George's University School of Medicine, St. George, GRD; 3 Internal Medicine, St. George's University School of Medicine, St. George, GRD; 4 Internal Medicine and Infectious Diseases, Wyckoff Heights Medical Center, Brooklyn, USA

**Keywords:** pyogenic liver abscess, streptococcus constellatus, streptococcus anginosus group, hepatology, infectious disease

## Abstract

Pyogenic liver abscess (PLA) is known as a pus-filled lesion found in the liver which can quickly become fatal if not found and treated in a timely manner. The most common group of bacteria found in PLA is the Streptococcus Anginosus Group (SAG). Patients with PLA usually present with fever and right upper quadrant abdominal pain which can at times be referred to the right shoulder owing to dermatomal involvement. We present a case where a patient with a past medical history significant for recent diverticulosis presenting with a left lower quadrant abdominal pain, fever, and hypotension and on further workup was found to have a PLA. Blood cultures and cultures from the abscess grew Streptococcus constellatus. This bacteria is part of the SAG group however, it is rarely found in PLA and bloodstream.

## Introduction

Liver abscess is defined as a pus-filled mass within the liver that can develop from direct injury or from an intra-abdominal infection which gets hematogenously seated via the blood supply from the portal vein. Most of these abscesses are pyogenic or amoebic, although a minority may be caused by parasites and fungi. If left untreated, deep-seated liver abscess can be a cause of severe morbidity and mortality. With time the abscess tends to become walled off and get further organized and may form septations within the wall which further complicates treatment. Many such complex abscesses become unresponsive to parenteral antibiotic treatment and require further surgical exploration and drainage, which in turn carry their own risks including but not limited to perforation and spillage of the contents in the intra-abdominal cavity, anaphylaxis, subphrenic abscesses, fistulas, and even death.

## Case presentation

A 72-year-old female with a past medical history significant of recent diverticulitis (a couple of months prior to admission), transient ischemic attack, coronary artery disease, fibromyalgia, spinal fusion surgery, and anxiety disorder presented to our institution as febrile (Maximum temperature: 102 F), hypotensive, poor oral intake, left lower quadrant abdominal pain and an episode of non-bloody, non-bilious vomiting. The patient was initiated on ceftriaxone 1 gram and metronidazole 500 mg every 8 hours intravenously due to her recent history of diverticulitis.

Upon admission, the patient’s laboratory values were significant for leukocytosis of 18.10 K/UL (4.5 - 10.9 K/UL), neutrophil 90.2%, hemoglobin 11.1 g/dl (12.2-15.0 g/dl), and platelets count 184 K/uL (130-400 K/uL). Lactic acid was elevated to 2.3 (0.4-2.0 mmol/L). Liver enzyme tests were significant for mildly elevated aspartate aminotransferase of 68 (15-37 U/L), alanine aminotransferase of 77 (12-78 U/L), and alkaline phosphatase (ALP) of 152 (45-117 U/L). Blood cultures were positive for Streptococcus constellatus and urine culture was negative.

**Figure 1:**
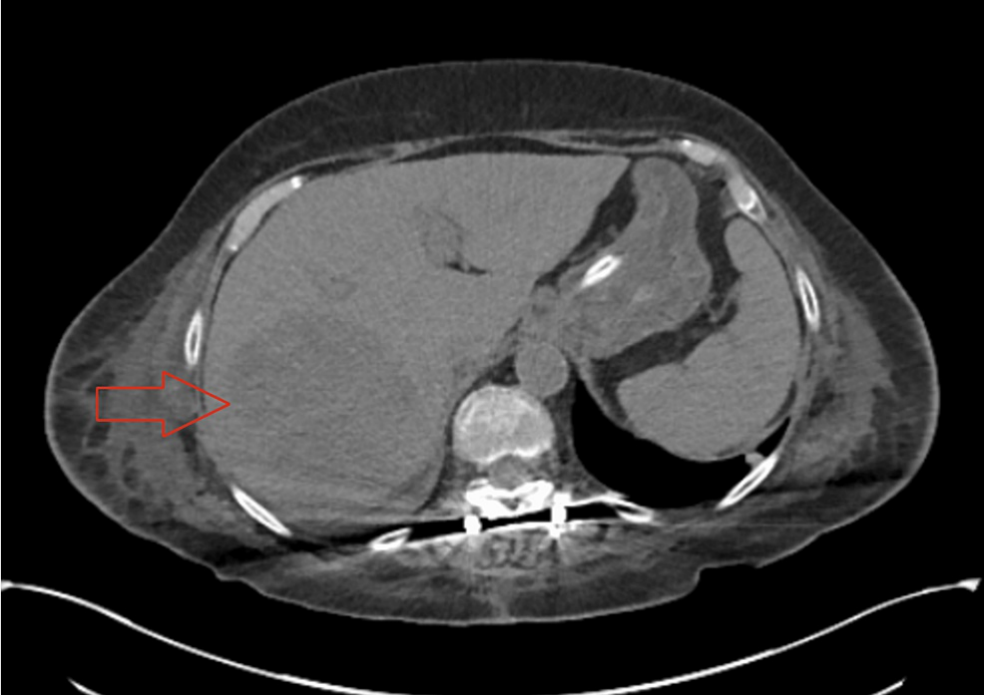
Multiple liver lesions are present on the CT Abdomen of the axial section. The largest occupies a significant portion of the right lobe of the liver and if it does not represent two lesions adjacent to one another measures overall close to 9.4 cm. The contour of the liver is mildly irregular.

**Figure 2:**
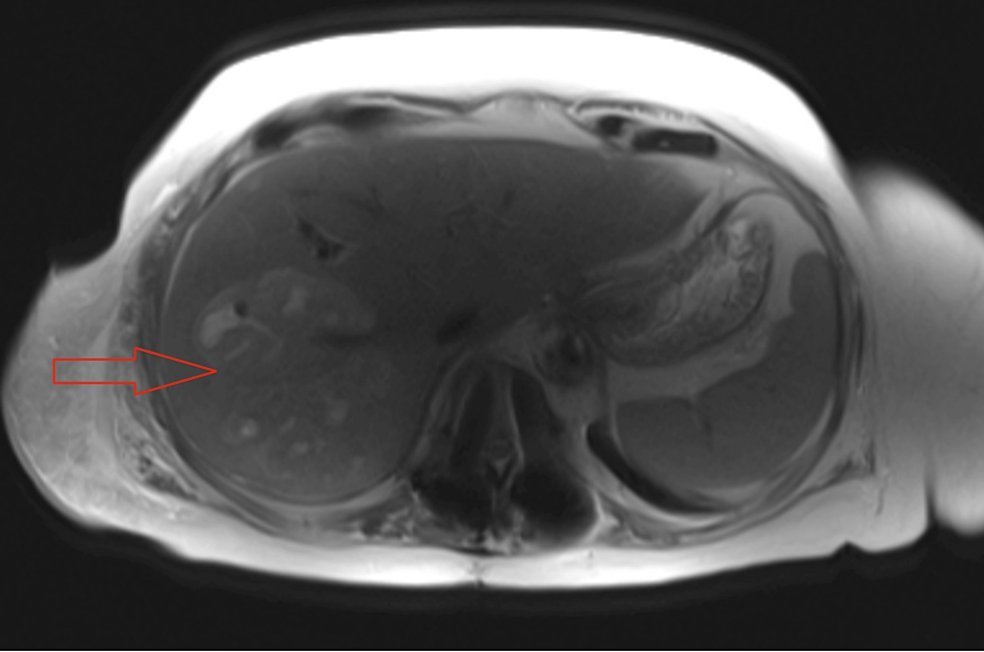
Multiple liver lesions are present on the MRI of the abdomen with contrast axial section.

On hospital day 5, the patient started complaining of right shoulder pain. In order to further investigate the cause of the right shoulder pain a computer tomography scan (CT scan) of the abdomen and pelvis was done. The reading was only significant for very mild inflammatory changes adjacent to the colon in the left lower quadrant, and mild diverticulitis. However, there was a hypodense lesion in the CT scan of the abdomen and pelvis (Figure 1). Interventional radiology was consulted for an image-guided biopsy of the hypodense lesion in the liver, which resulted in a rare gram-positive cocci (Streptococcus constellatus). Magnetic resonance imaging of the abdomen conveyed multiple hepatic heterogeneous and complex lesions that interrupted normal vascular branching patterns (Figure 2). General surgery was consulted for the exploration of the liver lesion. Metronidazole and ceftriaxone were discontinued and the patient was started on Meropenem 1gm IV every 8 hours.

Intensive care consult was sought for possible transfer after the surgical intervention. The patient underwent a diagnostic laparoscopy, intraoperative ultrasound-guided incision and drainage, washout of subhepatic abscess, and hepatic biopsy. The patient tolerated the surgery well and was transferred to the intermediate care unit postoperative day 2 and the patient was started on oral feeds and was noted to tolerate diet well. The surgical biopsy of the liver conveyed hepatic parenchyma with necrosis consistent with abscess. The patient had a peripherally inserted central catheter placed in order to obtain Meropenem 1 gram daily for a total of six weeks. The patient was safely discharged home with infusion services.

## Discussion

Pyogenic liver abscesses (PLA) cause approximately 15 of 100,000 hospital admissions, with an estimated incidence of 0.5-0.8% [1]. Albeit rare, they can become life-threatening with a 100% mortality rate without timely diagnosis and intervention. Once treated, the mortality rate is between 2.5-14% [2]. PLA tends to have polymicrobial etiology including Klebsiella (21%), E. Coli (16%), and Streptococcus Anginosus Group (SAG) (25%) [3]. A retrospective cohort study conducted from 2009-2015 concluded that intra-abdominal abscesses secondary to SAG were identified in 160/263 patients (60%) [4]. Another retrospective analysis (2014-2019) highlighted the prevalence of infection among the SAG species; of the 463 samples, 173 were attributed to S. constellatus (37.37%) [5]. Therefore, it is plausible to reason that S. constellatus and other bacteria belonging to SAG should be included when evaluating the etiology of PLA.

This group of microorganisms also causes suppurative infections in other locations such as the lung and brain [6]. In a retrospective study, among 30 patients with a respiratory infection (abscess, pneumonia, pleurisy) S. constellatus was identified in 11 (36.7%) of patients [7]. The liver (particularly the right lobe which is affected in 91% of cases) has a dual blood supply and is therefore susceptible to the formation of PLA due to disseminated sepsis by means of the hepatic artery or by translocation of a gastrointestinal infection via the portal system [8-9]. Insults to the gastrointestinal mucosa can mediate the entry of the organism through the intestinal mucosa and into the bloodstream with subsequent translocation to the liver [10]. A retrospective study showed that in patients with colonic diverticular disease (diverticulosis/diverticulitis), the incidence of PLA is 2.44 times higher than patients without diverticular disease [11]. This is also consistent with the findings of another study which concluded that colonic diverticulitis was associated with 27.5% of cases of PLA [12]. A review of the literature revealed other risk factors include biliary tree malignancies, particularly cholangiocarcinoma and hepatobiliary infections which may also lead to SAG-related liver abscess [13]. Dental procedures can also result in a temporary bacteremia, leading to the spread of the bacteria to the liver [14]. Other factors that increase the risk of SAG infection include chronic diseases (52% of patients) such as type 2 diabetes, hypertension, cancer, and smoking (33%) [15]. Based on previous literature, age (>65) also has a role in increasing the mortality rate in patients with SAG infections [16]. In this case, the patient had various risk factors for the development of S. constellatus bacteremia with translocation to the liver, including underlying diverticular disease and age >65.

The clinical presentation of PLA varies depending on the size and location of the abscess. The typical presentation includes abdominal pain, fever with other common symptoms including nausea, vomiting, weight loss, and malaise. These symptoms are quite vague and can point to numerous various pathologies. Although rare, respiratory symptoms secondary to the formation of pleural effusions may also result. The most common signs are guarding upon palpation of the right hypochondrium and high fevers [8]. In a retrospective study, abdominal pain and fever were present in 71% and 69%, respectively. CBC abnormalities typically included leukocytosis and elevated ALP and approximately 44% of patients had bacteremia [17]. This patient had a few signs and symptoms (fever, abdominal pain, and bacteremia) in common with the typical presentation of liver abscess; however, she did not have guarding in the right upper quadrant. A unique presentation in this case is right shoulder pain in the absence of right upper quadrant pain/guarding. This can possibly be explained by diaphragmatic irritation with referred pain to the right shoulder. Initially, the patient was worked up for diverticulitis as she had a medical history significant for recurrent diverticulitis. Our patient had a delay in diagnosis of PLA due to the absence of the right upper quadrant pain upon initial physical examination and the rarity of these cases. However, on hospital day 5, when she began complaining of right shoulder pain with intractable nausea/vomiting, imaging studies were probed for an explanation. Currently, the modality of choice for diagnosing PLA, whether amoebic or pyogenic, is the abdominal ultrasound, although CT scan is commonly used [18]. The solitary use of imaging is insufficient to distinguish the nature of the abscess. PLA warrants cultures for identification and sensitivity testing of culprit organisms which is obtained using image-guided biopsy/aspiration. This diagnostic step is crucial since it allows for appropriate management and treatment [19].

The treatment of PLA includes drainage and antibiotic therapy. The method of drainage is heavily dependent upon the size and accessibility of the abscess while the empiric antibiotic therapy is guided by the most probable source of infection until identification and sensitivity testing is performed. Then, the antibiotic regimen can be adjusted to cover targeted organisms. The first-line treatment of PLA 5-7.3 cm in diameter in patients having sustained fevers for >24-48 hours is image-guided percutaneous drainage along with systemic antibiotics [20]. For PLA >7.3 cm, the preferred option is surgical drainage as these have a higher tendency to be multiloculated with an increased risk of spontaneous rupture [2]. The first-line empiric antibiotic regimen includes a third-generation cephalosporin in combination with metronidazole. In light of this patient’s recent history of diverticular disease, she was started empirically on ceftriaxone 1 gram and metronidazole 500 mg every 8 hours intravenously. However, once the culture results of the sample taken by interventional radiology returned, treatment was tailored to target the rare S. constellatus identified. Diagnostic laparoscopy with drainage was also preferred in this patient owing to the presence of multiple complex lesions. The patient tolerated the procedure well and was discharged with a central catheter to receive Meropenem 1g for six weeks. It is crucial to consider the presence of SAG organisms in cases involving hepatic lesions with concurrent sepsis. Patients should be initially started on a broad-spectrum antibiotic until the true bacterial culprit is identified. Physicians must consider a hepatic lesion once S. constellatus is found in the blood. In this case, due to her spinal fusion surgery, the hepatic lesions were not visibly prominent. However, once the blood culture grew S. constellatus, immediate surgical consultation and intervention was done.

## Conclusions

Although rare, SAG organisms must be considered when evaluating the source of PLA. There are numerous risk factors that can predispose patients to develop PLA involving SAG of which, especially diverticular disease. While PLA tends to present with right upper quadrant pain/guarding, it can also atypically present with only right shoulder pain. Imaging studies (ultrasound/CT scan) can be used to make the diagnosis of PLA however, it must be followed up with drainage and cultures for targeted treatment. Patients can be placed on empiric antibiotic therapy until sensitivity testing can be performed. Once SAG bacteremia is identified, it is reasonable to consider the possibility of a concurrent presence of hepatic lesions.
